# Development and Psychometric Validation of the Hospital Medication System Safety Assessment Questionnaire

**DOI:** 10.3390/nursrep16010022

**Published:** 2026-01-12

**Authors:** Leila Sales, Ana Filipa Cardoso, Beatriz Araújo, Élvio Jesus

**Affiliations:** 1 Portuguese Red Cross Higher School of Health-Lisbon, Centre for Interdisciplinary Health Research (CIIS), Health Sciences Research Unit, Nursing, Nursing School of Coimbra, 3004-011 Coimbra, Portugal; 2 Health Sciences Research Unit, Nursing, Nursing School of Coimbra, 3004-011 Coimbra, Portugal; fcardoso@esenfc.pt; 3 Center for Interdisciplinary Research in Health (CIIS), Faculty of Health Sciences and Nursing, Universidade Católica Portuguesa, 4169-005 Porto, Portugal; baraujo@ucp.pt (B.A.); ejesus@ucp.pt (É.J.)

**Keywords:** patient safety, risk management, health care management, psychometrics, surveys and questionnaires, nursing

## Abstract

**Background/Objectives:** Medication incidents remain a significant concern in hospital settings. Integrated medication systems, regarding organized processes, policies, technologies and professional practices are designed to enhance patient safety; however, their safety performance is still suboptimal. The use of valid and reliable instruments to assess hospital medication system safety can be a valuable resource for health care management. The aim of this study was to describe the development and psychometric validation of the Hospital Medication System Safety Assessment Questionnaire (HMSSA-Q) for assessing the safety of hospital medication systems and its processes in Portugal. **Methods:** The HMSSA-Q was developed through a literature review and two rounds of expert panel consultation. Following consensus, a pilot methodological study was conducted in 95 Portuguese hospitals. Construct validity was assessed using principal component factor analysis, and reliability was evaluated through internal consistency (Cronbach’s alpha). **Results:** The instrument is theoretically structured into five predefined domains/subscales: Organizational Environment, Safe Medication Prescribing, Safe Medication in Hospital Pharmacy, Safe Medication Preparation and Administration, and Information and Patient Education. Principal component analyses performed separately for each domain supported their internal structure. The overall scale showed excellent internal consistency (Cronbach’s α = 0.97), with Cronbach’s alpha values for the domains ranging from 0.86 to 0.94. **Conclusions:** The HMSSA-Q is a valid and reliable instrument for assessing the safety of hospital medication systems and has the potential to serve as an innovative management tool for improving patient safety.

## 1. Introduction

Patient safety remains a global health priority because a substantial proportion of patients experience harm associated with healthcare delivery, with marked clinical and economic consequences [1,2]. Medication safety is a particularly complex domain of patient safety given the multi-step, multi-professional nature of medication uses within hospitals. Despite sustained international efforts to improve safety, preventable harm continues to occur at concerning levels across healthcare settings. In recognition of this burden, the World Health Organization (WHO) launched the third Global Patient Safety Challenge, *Medication Without Harm* and the Global Patient Safety Action Plan 2021–2030, calling for coordinated strategies to reduce severe, avoidable medication related harm [2,3]. Although preventable patient harm persists at meaningful levels, according to WHO data, worldwide, 1 in 10 patients suffers an adverse event while receiving hospital care, and 4 in 10 people suffer harm in home, primary, or community care [2]. A large meta-analysis estimated that preventable patient harm occurs in roughly 6% of patients across care settings, with medication incidents among the most frequent causes [4]. In the United States USA), a review reported that 25% of hospitalized Medicare beneficiaries experienced harm during a single month in 2018, and 43% of these events were considered preventable [5]. European policy statements similarly emphasize the scale and costs of adverse events and the need for system-level solutions [1]. Given the complexity and scope of the hospital medication, it is essential to adopt preventive strategies for medication errors and effective approaches to improving medication management safety that are transversal to all medication system processes, all professionals involved, and patients, and that are implemented at all levels of the institution. According to the Institute for Safe Medication Practices (ISMP), these strategies can be grouped into two main categories: (i) strategies focused on increasing system reliability, such as forcing functions, safety barriers and safeguards, computerization and automation, protocols and standardization of procedures, redundancies and duplicate checks, and alerts or checklists; and (ii) strategies focused on improving human reliability, including policies and rules, educational programs, provision of information, and precautionary recommendations. Although system focused strategies are more difficult to implement, they are generally more effective [6].

Hospital Medication System can be defined as a complex, organized and multiprofessional system with interdependent patient focused processes, that share the common goal of safely, effectively, appropriately and efficiently providing medicines to patients, involving doctors, pharmacists and nurses, patients and relatives/caregivers, with multiple stages and multiple passages of responsibility, influenced by the organizational and care environment [7]. Hospital medication systems encompass interdependent processes, from selection and procurement to storage, prescribing, transcription/verification, preparation and dispensing, administration, and monitoring, shaped by organizational context and involving multiple professional groups (e.g., pharmacists, physicians and nurses). Weaknesses at any process can propagate risk across the system [1,3].

Accordingly, assessing the safety of the system, rather than focusing solely on outcomes, aligns with the structure–process–outcome paradigm and supports proactive risk management and quality improvement [1,8]. The implementation of a broad and complementary set of strategies is essential to reduce the occurrence of medication errors and to enhance medication management safety in hospital settings. Given the complexity of the medication system, which involves multiprofessional teams, effective improvement strategies are largely grounded in the development and consolidation of a patient safety culture within healthcare organizations [3].

Methodological debates continue regarding how best to measure harm: incident reporting markedly under detects events compared with trigger-tool approaches such as the Institute for Healthcare Improvement (IHI) Global Trigger Tool, which has been shown to identify many more adverse events than conventional methods [9,10]. With regard to incident reporting systems, it is well established that continuous learning through spontaneous incident reporting is essential in this field, and that the reporting of medication safety incidents constitutes a tool that can contribute to the implementation of medication error prevention measures [7]. When adverse event reporting is conducted through electronic systems, it enables greater efficiency in communication processes and data retrieval, thereby facilitating monitoring and control of adverse event incidence [10]. In addition, hospital accreditation programs encourage institutions to strengthen incident reporting practices, analyze reported events, and implement preventive measures [11].

To achieve meaningful gains in patient safety, it is crucial to conduct a detailed assessment of each healthcare organization and to invest in areas identified as most vulnerable. These include, for example, the development and implementation of policies and procedures, continuous education and training related to medication use, effective communication, adequate working conditions, computerized prescribing systems, and advanced technologies for medication administration [12]. Such initiatives should subsequently be adapted and reflected across the different services of the institution, taking into account their specific characteristics and incident reporting systems. Although guidance and self-assessment checklists exist (e.g., ISMP Medication Safety Self Assessment^®^ and Targeted Medication Safety Best Practices), there is a relative shortage of psychometrically validated instruments that quantify the safety of hospital medication systems and enable benchmarking across organizations and over time [13,14]. Heterogeneity in definitions (e.g., medication error vs. adverse drug event vs. adverse drug reaction), measurement strategies (self-reporting vs. active surveillance), and case-mix further complicates comparisons across studies and underscores the need for robust, validated measurement tools [13,14,15], appropriate for the European healthcare context.

Objective. This study addresses these gaps by developing and psychometric validation of the Hospital Medication System Safety Assessment Questionnaire (HMSSA-Q) for assessing the safety of hospital medication systems and its processes in Portugal. In brief, the study provides initial evidence supporting construct validity and internal consistency of the instrument, offering a standardized measure to guide organizational learning and improvement.

## 2. Materials and Methods

### 2.1. Study Design

A methodological study was conducted to develop and validate the Hospital Medication System Safety Assessment Questionnaire (HMSSA-Q), involving the development and psychometric validation of a questionnaire, including item generation, content validation, pre-testing, and assessment of construct validity and reliability. The study comprised two phases: Phase I focused on the construction and content validation of the instrument, and Phase II assessed its psychometric properties.

### 2.2. Instrument Development

Prior to the study, an extensive literature review was undertaken to define the conceptual framework and select theoretical references, targeting patient safety and medication safety. Searches were conducted in scientific databases and documents from international organizations dedicated to these domains. Based on the literature review and the absence of an existing tool meeting the study’s objectives, a new instrument was developed following seven stages [16]: (1) definition of the conceptual structure; (2) definition of objectives and target population; (3) construction of items and response scales; (4) selection and organization of items; (5) instrument structuring; (6) content validity; (7) pre-testing.

The conceptual framework was defined using literature, guidelines, and expert consultation to establish the instrument’s scope, target population, and domains. Items were constructed and organized according to clarity, objectivity, and coverage of the Hospital Medication System definition, its attributes, and processes [16]. A 4-point Likert scale (1 = Does not exist/No implementation planned, 2 = Does not exist/Implementation planned within 3 to 6 months, 3 = Exists/Implemented in some departments, 4 = Exists/Implemented throughout the institution) was selected for responses.

### 2.3. Content Validation

Content validity was assessed via a Delphi technique with a multidisciplinary panel of 10 experts (The panel included representatives from the four main professional groups involved in hospital medication systems: hospital administrators, physicians, nurses, and pharmacists), selected based on predefined criteria, including clinical experience, research history about the topic, and expertise in patient safety, risk management, and instruments development. All panel members had demonstrated experience in areas such as healthcare management, clinical practice, and academia. Experts were invited via email, provided with study objectives, the instrument, and an evaluation grid for item relevance (1 = not relevant, 4 = extremely relevant). After the first round, results and comments were summarized and of the 187 items submitted for consensus, adaptations were suggested for 32 items in order to improve their suitability and clarity. The construct was subsequently revised and resubmitted in a second Delphi round to ensure that it did not present comprehension difficulties. After completion of the second-round questionnaires, and given that a level of consensus exceeding 75% was achieved among the experts for all items, a third round was deemed unnecessary. Consensus ≥ 75% was generally accepted in the literature [16,17], but given the high agreement rates from the start, only items with ≥90% agreement were retained. Following expert feedback, items were revised or removed, yielding the final pre-test version.

### 2.4. Pre-Testing

The pre-test aimed to ensure semantic clarity and feasibility. It was applied to a convenience sample of 10 former members of hospital risk management teams. Participants assessed clarity, item relevance, and completion time. Minor adjustments were made, resulting in the final instrument ready for application to the study population.

### 2.5. Sample and Data Collection

The study targeted all Portuguese hospitals (N = 118), regardless of legal or administrative status. A total of 95 hospitals (*n* = 95) participated in the study, corresponding to 95 completed questionnaires. The unit of analysis was the hospital. Given the scope of the questionnaire and the type of information collected, each questionnaire was completed by the hospital’s Quality and Safety Committee (QSC), ensuring a multiprofessional assessment. The QSCs typically include healthcare professionals with expertise in medication management and patient safety, such as nurses, pharmacists, and physicians. The consensus was reached by deliberation meetings and in the end the chair of the QSC submitted a consolidated response. Data were collected online from November of 2019 until December of 2020 using Qualtrics Survey^®^, with restricted access through unique links for each hospital, ensuring privacy and data security.

### 2.6. Instrument, Variables and Measures

The HMSSA-Q includes a section for hospital characterization; (1) Geographical location; (2) Organizational structure; (3) Management model and legal status; (4) Teaching status; (5) Type of hospital care; (6) Number of inpatient beds; (7) Existence of a Health Quality and Accreditation System and (8) Accreditation program. Three closed questions (Does the institution have a risk management/patient safety team? Is medication safety one of the institution’s priorities? Please assign your institution a level related to the safety of the medication system (5 Excellent, 4 Very good, 3 Acceptable, 2 Poor, 1 Very poor), and five subscales covering:

(1) Medication safety and organizational environment;
(2) Safety in medication prescribing;
(3) Medication safety in hospital pharmacy;
(4) Safety in medication preparation and administration;
(5) Medication safety in patient information and education.

### 2.7. Psychometric and Statistical Analysis

Psychometric properties were assessed using IBM SPSS Statistics version 28. Analyses included descriptive statistics, Pearson correlations for item homogeneity, Cronbach’s alpha for internal consistency, and principal component analysis (PCA) for construct validity. PCA used principal component analysis with varimax rotation, eigenvalues ≥ 1, and factor loadings ≥ 0.40. Factor retention was primarily guided by the Kaiser criterion (eigenvalue ≥ 1), in conjunction with the underlying theoretical model and strict criteria for item loadings and cross-loadings. Accordingly, the extracted component structures should be interpreted as exploratory.

### 2.8. Ethical Considerations

The study was approved by the Ethics Committee of the Universidade Católica Portuguesa of Lisbon, Portugal (decision number 35-CE-2017). All ethical and legal principles were met. The study was conducted in accordance with the Declaration of Helsinki. Participation was voluntary, and information about the purpose and nature of the study was given to all participants that signed an informed consent form.

## 3. Results

### 3.1. Instrument Development Outcomes

The development and psychometric evaluation of the HMSSA-Q followed a sequential, multi-stage process of item reduction and dimensional analysis. An initial pool of 193 items was generated based on an extensive review of the literature on medication safety systems and relevant international guidelines. Prior to the Delphi rounds, six items were merged and removed due to redundancy and semantic overlap identified during an internal expert review. This resulted in a refined set of 187 items that were subsequently evaluated by the Delphi panel. In the first stage, content validity was assessed using a Delphi technique with an expert panel. Items were reviewed in terms of relevance, clarity, and redundancy, and decisions regarding item elimination or reformulation were based on expert agreement. This process resulted in a reduced set of 115 items retained for psychometric testing.

### 3.2. Content Validity Results

The Delphi panel (*n* = 10 experts) reached high consensus levels, with an average Scale Content Validity Index (S-CVI/ave) of 0.92 across all dimensions, exceeding the recommended threshold of 0.90 for new scales [16,17]. While a minimum consensus threshold of ≥75% agreement was considered acceptable for identifying items requiring revision, only items achieving ≥90% agreement were retained without modification. Items with agreement between 75% and 89% were revised based on expert feedback and re-evaluated in subsequent Delphi rounds, whereas items below 75% were removed. Items were reduced to 115 after the Delphi rounds.

### 3.3. Factor Analysis Results

To assess the psychometric properties of the Hospital Medication System Safety Assessment Questionnaire (HMSSA-Q), we examined its dimensionality and internal consistency. Dimensionality was evaluated using PCA with orthogonal varimax rotation, and internal consistency was assessed using Cronbach’s alpha coefficient. An initial exploratory PCA was conducted within each predefined domain, rather than on the full item set, in accordance with the theoretical model underlying the instrument. Each domain represents a distinct construct, and therefore the correlation structure was considered meaningful within, but not necessarily across, domains. For each PCA, component retention was based on the eigenvalue ≥ 1 criterion, and items were required to display primary loadings ≥ 0.40 and no cross-loadings within 0.20 of the primary loading, in line with established methodological recommendations [18].

Across the five domains, the PCA supported the proposed internal structures, yielding 4 components for Organizational Environment (MSOE), 3 for Safe Medication Prescribing (SMP), 4 for Safe Medication in Hospital Pharmacy (MSHP), 4 for Safe Medication Preparation and Administration (SMPA), and a unidimensional structure for Information and Patient Education (MSPIE). Items not meeting the predefined criteria were excluded, resulting in a final instrument comprising 97 items. Sampling adequacy for factor analysis was confirmed for all domains. The Kaiser–Meyer–Olkin (KMO) measures ranged from 0.685 to 0.826, and Bartlett’s tests of sphericity were statistically significant for all analyses (*p* < 0.001), indicating that the correlation matrices were suitable for PCA.

The resulting component structures demonstrated coherent item groupings that were conceptually aligned with the theoretical framework of each domain. Factor loadings and communalities for the retained items are presented in Table 1, and Table 2 summarizes the distribution of items across domains and their respective components. Reliability indices for each domain are reported below.

### 3.4. Internal Consistency

The internal consistency of each subscale, as measured by Cronbach’s alpha, was assessed immediately after confirming the final factor structure. This allowed the evaluation of the reliability of the factor solution identified in the PCA. The HMSSA-Q demonstrated excellent internal consistency, with an overall Cronbach’s alpha of 0.97. Subscale alphas ranged from 0.80 to 0.95. Detailed subscale analyses are presented in Table 3, Table 4, Table 5, Table 6, Table 7, Table 8, Table 9, Table 10, Table 11 and Table 12.

### 3.5. Medication Safety and Organizational Environment Subscale

The dimensionality of the Medication Safety and Organizational Environment (MSOE) subscale was assessed using PCA with orthogonal varimax rotation. Sampling adequacy and the suitability of the correlation matrix were confirmed (Kaiser–Meyer–Olkin = 0.824; Bartlett’s test of sphericity = 1691.916, *p* < 0.001). Four components with eigenvalues ≥ 1 were extracted, accounting for 67.37% of the total variance (Table 3). Item retention was based on factor loadings ≥ 0.40. Communalities were generally satisfactory (>0.40). The four components were identified as:

1. Safe Practices (44.83% of the variance; eigenvalue = 10.31; Cronbach’s α = 0.95),
2. Management of LASA/High-Alert Medications and Incident Reporting (8.74%; eigenvalue = 2.01; α = 0.83),
3. Training and Medication Reconciliation (7.16%; eigenvalue = 1.65; α = 0.84), and
4. Policies and Procedures (6.63%; eigenvalue = 1.53; α = 0.84).

Internal consistency for the 23-item subscale was excellent (Cronbach’s α = 0.94), well above the recommended minimum of 0.70, indicating strong inter-item correlation and homogeneity. Corrected item–total correlations ranged from 0.46 to 0.80, all exceeding the accepted threshold of 0.20. The mean scores for individual items were generally around the midpoint of the scale (2.20 to 3.80), with standard deviations between 0.59 and 1.35.

Descriptive statistics for each factor are summarized in Table 4. The highest mean score was observed for Policies and Procedures (mean = 3.49, SD = 0.70), followed by Management of LASA/High-Alert Medications and Incident Reporting (mean = 3.15, SD = 0.73) and Safe Practices (mean = 3.09, SD = 0.82). The lowest mean was for Training and Medication Reconciliation (mean = 2.56, SD = 0.96). Across the 95 hospitals, the overall mean score for the MSOE subscale was 3.10 (SD = 0.68; range 1.70–4.00), with 58.4% of ratings between 3 and 4, 34.7% between 2 and 3, 6.3% between 1 and 2, and 2.1% at the maximum value of 4.

### 3.6. Medication Prescription Safety Subscale

The dimensionality of the Medication Prescription Safety (MPS) subscale was assessed using principal component analysis (PCA) with orthogonal varimax rotation. Sampling adequacy and the suitability of the correlation matrix were confirmed (Kaiser–Meyer–Olkin = 0.727; Bartlett’s test of sphericity = 712.653, *p* < 0.001). Three components with eigenvalues ≥ 1 were extracted, explaining 77.58% of the total variance (Table 5). Item retention was based on factor loadings ≥ 0.40.

The three components were identified as:

1. Policies, Procedures, and Safe Practices (PPPS) (47.87% of variance; eigenvalue = 5.56; Cronbach’s α = 0.81),
2. Electronic Prescription (PE) (14.17% of variance; eigenvalue = 2.31; α = 0.75) and
3. Communication and Information Management (CIM) (8.24% of variance; eigenvalue = 1.68; α = 0.80).

Communalities were generally satisfactory (>0.40) for most items. The internal consistency of the total 18-item subscale was good (Cronbach’s α = 0.86), with corrected item–total correlations ranging from 0.23 to 0.74.

Descriptive statistics for each factor are presented in Table 6. Mean scores ranged from 2.42 (SD = 0.76–0.95) for PPPS and PE to 3.05 (SD = 0.86) for CIM. Across the 95 hospitals, the mean total SMP score was 2.62 (SD = 0.65; range 1.28–4.00), with 47.4% of responses between 2 and 3, 34.7% between 3 and 4, 15.8% between 1 and 2, and 2.1% at the maximum value of 4.

### 3.7. Medication Safety in Hospital Pharmacy Subscale

The internal structure of the Medication Safety in Hospital Pharmacy (MSHP) subscale was examined using principal component analysis (PCA) with orthogonal varimax rotation. Sampling adequacy was confirmed by the Kaiser–Meyer–Olkin (KMO) measure (0.685) and Bartlett’s test of sphericity (=1278.331, *p* < 0.001), indicating the suitability of the correlation matrix for factor analysis. PCA identified a four-factor solution explaining 57.42% of the total variance (Table 7). Factor I (“Labeling and Storage”) accounted for 30.32% of the variance (eigenvalue = 7.28; α = 0.85), Factor II (“Training and Safe Practices”) for 11.01% (eigenvalue = 2.64; α = 0.77), Factor III (“Policies and Procedures”) for 8.63% (eigenvalue = 2.07; α = 0.70), and Factor IV (“Dispensing and Distribution”) for 7.45% (eigenvalue = 1.79; α = 0.79). Items retained had loadings above 0.40. Internal consistency analysis showed a Cronbach’s α of 0.87 for the overall subscale, classified as “very good” [16], and well above the recommended 0.70 threshold for such instruments [15]. Corrected item–total correlations ranged from 0.23 to 0.68, and mean item scores varied from 2.02 to 3.97 (scale range: 1–4), with standard deviations between 0.18 and 1.33.

Descriptive statistics by factor are presented in Table 8. The highest mean score was obtained for “Labeling and Storage” (M = 3.66, SD = 0.51) and “Dispensing and Distribution” (M = 3.66, SD = 0.55), while “Training and Safe Practices” scored lowest (M = 2.95, SD = 0.75). The overall MSHP mean score across the 95 hospitals was 3.28 (SD = 0.48; range: 2.17–4.00), with 75.8% of hospitals scoring between 3 and 4, and none scoring below 2.

### 3.8. Safety in Medication Preparation and Administration Subscale

The internal structure of the Safety in Medication Preparation and Administration (SMPA) subscale was evaluated through principal component analysis (PCA) with orthogonal varimax rotation. Sampling adequacy was confirmed by the Kaiser–Meyer–Olkin (KMO) measure (0.695) and Bartlett’s test of sphericity (=1536.935, *p* < 0.001), indicating the suitability of the correlation matrix for factor analysis.

The analysis identified a four-factor solution explaining 56.23% of the total variance (Table 9). Factor I (“Policies and Procedures”) accounted for 26.30% of the variance (eigenvalue = 7.10; α = 0.87), Factor II (“Safe Practices”) for 11.25% (eigenvalue = 3.04; α = 0.88), Factor III (“Work Environment”) for 10.02% (eigenvalue = 2.70; α = 0.75), and Factor IV (“Available Resources”) for 8.66% (eigenvalue = 2.34; α = 0.70). All items presented factor loadings above 0.40. The overall internal consistency of the SMPA subscale was good (Cronbach’s α = 0.88), exceeding the recommended 0.70 threshold [15,16]. Corrected item–total correlations ranged from 0.26 to 0.67, and mean item scores varied from 2.02 to 3.73 (scale range: 1–4), with standard deviations between 0.63 and 1.40. Descriptive statistics per factor are summarized in Table 10. The highest mean score was observed for “Policies and Procedures” (M = 3.31, SD = 0.69) and the lowest for “Safe Practices” (M = 2.66, SD = 0.89). The overall SMPA mean score across 95 hospitals was 2.91 (SD = 0.56; range: 1.46–3.86), with 55.8% of hospitals scoring between 2 and 3, and 42.1% scoring exactly 3, while only 2.1% scored at the minimum level.

### 3.9. Medication Safety in Patient Information and Education Subscale

The Medication Safety in Patient Information and Education (MSPIE) subscale was analyzed for dimensionality and internal consistency. Sampling adequacy was confirmed by the Kaiser–Meyer–Olkin (KMO) measure (0.826) and Bartlett’s test of sphericity (=217.361, *p* < 0.001), supporting the suitability of the correlation matrix for factor analysis.

PCA revealed a unidimensional structure, with the five items explaining 64.06% of the total variance (Table 11). Factor loadings ranged from 0.63 to 0.88, with communalities above 0.40, except for one item at 0.40. The factor was labeled “Patient Information and Education” and demonstrated good internal consistency (Cronbach’s α = 0.85), exceeding the recommended threshold of 0.70 [15,16].

Corrected item–total correlations ranged from 0.49 to 0.79, and mean item scores varied between 2.08 and 2.71 (scale range: 1–4), with standard deviations between 1.14 and 1.33. The mean MSPIE score across 95 hospitals was 2.46 (SD = 0.99; range: 1.00–4.00), with 32.6% of hospitals scoring between 1 and 2, 30.6% between 2 and 3, 24.2% between 3 and 4, and 12.6% achieving the maximum score (Table 12).

## 4. Discussion

The present study developed and psychometrically validated the *Hospital Medication System Safety Assessment Questionnaire* (HMSSA-Q), a multidimensional tool addressing five critical domains of hospital medication safety: Medication Safety and Organizational Environment (MSOE), Safety in Medication Prescribing (SMP), Medication Safety in Hospital Pharmacy (MSHP), Safety in Medication Preparation and Administration (SMPA), and Medication Safety in Patient Information and Education (MSPIE) (Supplementary Materials).

The instrument demonstrated high content validity (S-CVI = 0.92), exceeding the widely accepted threshold of 0.80 for new scales [16,17], suggesting that the selected items adequately represent the construct of hospital medication system safety. The rigorous Delphi process ensured a high degree of expert consensus and contextual relevance, consistent with recommendations for scale adaptation to complex healthcare systems [19]).

PCA conducted separately within each subscale revealed clear and interpretable component structures, with acceptable sampling adequacy (KMO values ranging from 0.685 to 0.826) and highly significant Bartlett’s test results (*p* < 0.001), confirming the suitability of the data for factor analysis. The proportion of explained variance within subscales ranged from approximately 49% to over 77%, values that are comparable to or higher than those reported in similar psychometric studies of patient safety climate or medication safety culture instruments [20,21].

Internal consistency was excellent for the overall scale (Cronbach’s α = 0.97) and high across all subscales (α = 0.75–0.95), indicating strong homogeneity and reliability. The MSOE subscale, for instance, achieved α = 0.94, reflecting the interrelatedness of safe practice indicators such as high-alert medication management, incident reporting culture, and reconciliation processes. Similarly, the SMP subscale demonstrated robust reliability (α = 0.86) despite the diversity of its domains (policies, electronic prescribing, and communication strategies), underlining the coherence of the prescribing safety construct. The overall Cronbach’s alpha observed for the HMSSA-Q was very high, reflecting a strong internal consistency across items. While high alpha values may, in some cases, suggest item redundancy, the present findings should be interpreted in light of the multidimensional and system-level nature of the instrument, which was designed to comprehensively capture multiple domains of medication safety. Item–total correlations and alpha-if-item-deleted analyses supported the internal coherence of the scale and did not indicate clear advantages in removing individual items at this stage. Nevertheless, the length of the questionnaire may represent a feasibility consideration in hospital settings. Accordingly, future research should focus on further psychometric refinement, including the use of complementary reliability indices and targeted item reduction strategies, with the aim of developing shorter versions of the instrument while preserving its conceptual breadth and measurement robustness.

The distribution of scores across hospitals provided important insights into the implementation level of safety practices. Higher median scores in Policies & Procedures (MSOE Factor IV) and Communication & Information Management (SMP Factor III) indicate that formal documentation and structured communication are relatively well-established. Conversely, lower mean scores in Training & Medication Reconciliation (MSOE Factor III) and Policies, Procedures & Safe Practices in prescribing (SMP Factor I) highlight potential areas for improvement, particularly in proactive risk prevention and interprofessional coordination.

These findings align with previous reports identifying medication reconciliation and integration of clinical decision support as persistent weak points in hospital medication safety systems [22,23,24]. A combination of individual level measures such as educational interventions for professionals, system level measures related to work environments, and organizational level measures such as the introduction of quality monitoring and improvement processes are coordinated and promising strategies for mitigating preventable harm to patients [4].

From a practical standpoint, the HMSSA-Q offers a comprehensive and context-sensitive diagnostic framework for hospital administrators, patient safety officers, doctors, nurses and clinical pharmacists. By breaking down medication safety into granular, measurable components, the tool facilitates targeted interventions and benchmarking over time, potentially complementing broader safety climate surveys and accreditation audits.

However, some limitations warrant consideration. First in this exploratory phase, an orthogonal (varimax) rotation was used in the principal component analyses to support a clear and interpretable structure aligned with the predefined domains of the instrument and its intended institutional application. While this approach assumes independence among components, it was considered appropriate for an initial examination of the questionnaire’s internal structure. Future studies, using larger samples and multiple respondents per institution, may further explore the dimensional relationships underlying medication safety by applying common factor extraction methods, oblique rotations, and confirmatory factor analysis, thereby contributing to the continued refinement of the instrument. While the sample size met the minimum requirements for PCA, a larger and more diverse hospital cohort could enhance the generalizability of factor structures, although in the Portuguese context we had reach 80.59% of the study population. Also factor retention relied primarily on the Kaiser criterion (eigenvalue ≥ 1). Although widely used, this approach has well-documented limitations when applied as the sole retention method. Consequently, the factor structures identified in this study should be considered preliminary and warrant confirmation in future studies using more robust retention techniques and confirmatory factor analysis.

Second, the cross-sectional design precludes assessment of test–retest reliability and sensitivity to change over time. Third, the reliance on self-reported data may introduce social desirability bias, particularly in domains linked to regulatory compliance.

Future research should aim to validate the instrument in different healthcare contexts (e.g., primary health care, long-term care facilities) and in other languages through forward–backward translation. Assess predictive validity, examining whether higher HMSSA-Q scores correlate with lower rates of medication errors and adverse drug events and incorporate longitudinal design to evaluate responsiveness to safety interventions and health policy changes.

## 5. Conclusions

This study resulted in the development and psychometric validation of the *Hospital* Medication System Safety Assessment Questionnaire (HMSSA-Q), a novel, multidimensional instrument designed to evaluate key domains of medication safety within hospital settings. The rigorous methodological process, combining literature review, expert consensus via Delphi rounds, and principal component factor analysis ensured strong content validity, a robust factor structure, and high internal consistency across all subscales.

The findings highlight both strengths and opportunities within participating hospitals. Higher performance was observed in areas related to formal policies, procedures, and structured communication, whereas gaps were more evident in medication reconciliation, training, and integration of electronic safety mechanisms. These patterns are consistent with the international literature, reinforcing the need for targeted interventions in these domains.

By offering a comprehensive, context-sensitive framework, the HMSSA-Q can support healthcare organizations in systematically diagnosing safety strengths and weaknesses, benchmarking across facilities, and guiding strategic improvements. Its adaptability also allows potential use in other healthcare contexts and international settings, following cultural and linguistic validation.

Future work should focus on expanding the instrument’s application to diverse healthcare systems, assessing its predictive validity against medication error rates, and testing its sensitivity to change following safety interventions. The HMSSA-Q thus represents not only a measurement tool, but also a catalyst for continuous quality improvement and enhanced patient safety in medication management.

The HMSSA-Q is a psychometrically valid, multidimensional instrument that can support healthcare organizations in systematically assessing and improving medication safety. By providing a structured lens on organizational practices, prescribing, pharmacy operations, administration, and patient education, it addresses a critical gap in patient safety measurement and offers a practical roadmap for safer medication systems.

## Figures and Tables

**Table 1:** Factor Analysis Results by Subscale of the Hospital Medication System Safety Assessment Questionnaire.

Subscale	No. Items	No. Factors	%Variance Explained	Factors and Item Numbers
Medication Safety and Organizational Environment	23	4	67.37%	Factor I (12,15,16,18,19); Factor II (11,13,14,17,21,22,23); Factor III (1,7,8,9,20); Factor IV (2,3,4,5,6,10)
Safety in Medication Prescribing	18	3	52.98%	Factor I (30,37,39,40,41); Factor II (29,31,32,33,34,35,36); Factor III (24,25,26,27,28,38)
Medication Safety in Hospital Pharmacy	24	4	57.42%	Factor I (43,44,46,47,48,51,55,57,58); Factor II (52,53,54,59,60,65); Factor III (49,50,56,61,62,63,64); Factor IV (42,45)
Safety in Medication Preparation and Administration	27	4	56.23%	Factor I (69,70,71,75,84,85,89,90,91); Factor II (66,78,79,80,81,83); Factor III (67,68,72,74,86,87,88,92); Factor IV (73,76,77,82)
Medication Safety in Patient Information and Education	5	1	64.06%	Factor I (93,94,95,96,97)

**Table 2:** Subscales and Factors of the Hospital Medication System Safety Assessment Questionnaire.

Subscales	Factors
Medication Safety and Organizational Environment	− Policies and procedures − Safe practices − Management of LASA (Look-Alike, Sound-Alike)/high-alert medications and incident reporting − Training and medication reconciliation
Medication Prescription Safety	− Policies, procedures, and safe practices − Electronic prescribing − Communication and information management
Medication Safety in the Hospital Pharmacy	− Labeling and storage − Training and safe practices − Policies and procedures − Dispensing and distribution
Medication Preparation and Administration Safety	− Policies and procedures − Safe practices − Work environment − Available resources
Medication Safety in Patient Information and Education	− Patient information and education

**Table 3:** Factor analysis of the MSOE subscale and Cronbach’s alpha coefficients for each factor.

Factor	Variance Explained (%)	Eigenvalue	Cronbach’s α
Safe Practices	44.83	10.31	0.94
Management of LASA/High-Alert Medications and Incident Reporting	8.74	2.01	0.83
Training and Medication Reconciliation	7.16	1.65	0.84
Policies and Procedures	6.63	1.53	0.84

Note. LASA = Look-Alike, Sound-Alike.

**Table 4:** Internal consistency and descriptive statistics for the four factors of the MSOE subscale (n = 95).

Factor	n Items	Mean	SD	Median	Min	Max	Cronbach’s α
Safe Practices	5	3.09	0.82	3.38	1	4	0.94
Management of LASA/High-Alert Medications and Incident Reporting	7	3.15	0.73	3.33	1	4	0.83
Training and Medication Reconciliation	5	2.56	0.96	2.50	1	4	0.84
Policies and Procedures	6	3.49	0.70	4.00	2	4	0.84

Note. LASA = Look-Alike, Sound-Alike.

**Table 5:** Factor analysis of the SMP subscale and Cronbach’s alpha coefficients for each factor.

Factor	Variance Explained (%)	Eigenvalue	Cronbach’s α
Policies, Procedures, and Safe Practices (PPPS)	47.87	5.56	0.81
Electronic Prescription (PE)	14.17	2.31	0.75
Communication and Information Management (CIM)	8.24	1.68	0.80

**Table 6:** Descriptive statistics for the three factors of the SMP subscale (*n* = 95).

Factor	*n* Items	Mean	SD	Median	Min	Max	Cronbach’s α
Policies, Procedures, and Safe Practices (PPPS)	5	2.42	0.95	2.20	1	4	0.81
Electronic Prescription (PE)	7	2.42	0.76	2.29	1	4	0.75
Communication and Information Management (CIM)	6	3.05	0.86	3.33	1.33	4	0.80

**Table 7:** Factor analysis of the MSHP subscale and Cronbach’s alpha coefficients for each factor.

Factor	Variance Explained (%)	Eigenvalue	Cronbach’s α
Labeling and Storage (RA)	30.32	7.28	0.85
Training and Safe Practices (FPS)	11.01	2.64	0.77
Policies and Procedures (PP)	8.63	2.07	0.70
Dispensing and Distribution (DD)	7.45	1.79	0.79

**Table 8:** Descriptive statistics by factor of the MSHP subscale (*n* = 95).

Factor	Items (*n*)	Mean	SD	Min	Max	Cronbach’s α
RA	9	3.66	0.51	2.22	4.00	0.85
FPS	6	2.95	0.75	1.00	4.00	0.77
PP	7	2.99	0.64	1.67	4.00	0.70
DD	2	3.66	0.55	1.00	4.00	0.79

**Table 9:** Factor analysis of the SMPA subscale.

Factor	Variance Explained (%)	Eigenvalue	Cronbach’s α
Policies and Procedures (PP)	26.30	7.10	0.87
Safe Practices (PS)	11.25	3.04	0.88
Work Environment (AT)	10.02	2.70	0.75
Available Resources (RD)	8.66	2.34	0.69

**Table 10:** Descriptive statistics by factor of the SMPA subscale (*n* = 95).

Factor	Items (*n*)	Mean	SD	Min	Max	Cronbach’s α
PP	9	3.31	0.69	1.00	4.00	0.87
PS	6	2.66	0.89	1.00	4.00	0.88
AT	8	2.95	0.59	1.00	4.00	0.74
RD	4	2.85	0.87	1.00	4.00	0.69

**Table 11:** Factor analysis results for the MSPIE subscale.

Factor	Variance Explained (%)	Eigenvalue	Cronbach’s α
Patient Information and Education	64.06	3.20	0.85

**Table 12:** Descriptive statistics for the MSPIE subscale (*n* = 95).

Factor	Items (*n*)	Mean	SD	Min	Max	Cronbach’s α
PIE	5	2.46	0.99	1.00	4.00	0.85

## Data Availability

The data presented in this study are available on request from the corresponding author. The data are not publicly available due to the principal information and data are presented in this study, and more detailed information will be send to the readers after contacting the author so we can track the utilization of the questionnaire.

## Supplementary Materials

The following supporting information can be downloaded at: https://www.mdpi.com/article/10.3390/nursrep16010022/s1, Questionnaire for the Assessment of Hospital Medication System Safety (HMSSA-Q).

## Public Involvement Statement

There was no public involvement in any aspect of this research.

## Guidelines and Standards Statement

This manuscript was drafted against the STROBE checklist for cross-sectional studies research.
