# Supplementary material for: Development and Psychometric Validation of the Hospital Medication System Safety Assessment Questionnaire
Source: Nurs Rep. 2026 Jan 12;16(1):22. doi: 10.3390/nursrep16010022 (PMC12844372; doi:10.3390/nursrep16010022)
Supplement: Supplementary file 1 [file nursrep-16-00022-s001.zip › nursrep-4036674-supplementary.pdf]

QUESTIONNAIRE FOR THE ASSESSMENT OF HOSPITAL MEDICATION SYSTEM SAFETY (HMSSA- Q)

This questionnaire aims to assess the safety of the hospital medication system, in its multiple dimensions, in each hospital unit, in order to identify critical areas and those at higher risk for medication-related incidents.

Completion of this questionnaire is the responsibility of the Quality and Patient Safety Committee. All information is confidential.

Your collaboration is essential for understanding this reality.

CONCEPTS

Error – “A failure in the execution of a planned action as intended or the incorrect development of a plan.”

Patient Safety Incident – “An event or circumstance that could have resulted, or did result, in unnecessary harm to a patient.”

Adverse Event – “An incident that results in harm to the patient.”

Sentinel Event – “An unexpected occurrence involving death, serious physical or psychological injury, or the risk thereof.”

Medication Reconciliation – “The process of reviewing a patient’s medication whenever changes occur, with the aim of avoiding discrepancies, namely omissions, duplications, or inappropriate doses.”

HOSPITAL CHARACTERIZATION

1. Geographical country location (State/Region)

---

2. Legal status of the institution / 3. Management model (select all that apply):
☐ Hospital integrated into a Hospital Centre
☐ Hospital integrated into hospital groups
☐ Public Administrative Sector Hospital
☐ Public Business Entity Hospital
☐ Hospital integrated into a Local Health Unit
☐ Public-Private Partnership Hospital
☐ Private Hospital
☐ Other: ______________________

5. Teaching Hospital:
☐ Yes
☐ No

5. Type of Hospital Care:
☐ General Hospital
☐ Specialized Hospital – Main specialty: ______________________

6. Number of inpatient beds: ______________________

7. Quality System and Health Accreditation:
☐ Non-accredited institution
☐ Accredited services within the institution
☐ Accredited institution
☐ Institution undergoing accreditation
☐ No strategy implemented

8. Accreditation programme:
☐ CHKS
☐ Joint Commission International (JCI)
☐ Ministry of Health / ACSA International
☐ Other: ______________________

9. Does the institution have a risk management / patient safety team? ☐ Yes ☐ No

10. Is medication safety a priority in the institution? ☐ Yes ☐ No

11. Overall level of medication system safety:
☐ Excellent ☐ Very Good ☐ Acceptable ☐ Poor ☐ Very Poor

MEDICATION SAFETY AND ORGANIZATIONAL ENVIRONMENT SCALE

Response scale:
1 – Does not exist / Not planned
2 – Does not exist / Planned within 3–6 months
3 – Exists / Implemented in some services
4 – Exists / Implemented throughout the institution

**SAFE PRACTICES**				
	1	2	3	4
During medication procurement, does the institution consider alternatives, whenever possible, to avoid adding LASA medications to the internal list?				
Is the use of Tall Man lettering implemented to differentiate LASA medication names?				
Has the institution implemented training sessions on safe management of LASA medications?				
Is an institutional strategy implemented for the storage and identification of high-alert medications?				
Is patient safety prioritized when selecting materials and devices for medication preparation and administration?				
**MANAGEMENT OF LASA / HIGH-RISK MEDICATIONS AND INCIDENT REPORTING**				
	1	2	3	4
Does the institution have an updated and disseminated internal list of Look-Alike, Sound-Alike (LASA) medications?				
Do medication-related information systems include alerts for LASA medications?				
Is an institutional strategy implemented for the storage and identification of LASA medications?				
Does the institution have an updated internal list of high-alert medications?				
Are awareness campaigns conducted to encourage reporting of medication incidents or risk situations?				
Is it standardized that professionals must report all medication incidents they become aware of?				

Following the analysis of a serious adverse medication event or sentinel event, is information disseminated to all professionals?				
**TRAINING AND MEDICATION RECONCILIATION**				
	1	2	3	4
Is mandatory medication safety training provided to professionals involved in the medication system?				
Is there a procedure for performing medication reconciliation?				
Is medication reconciliation performed at admission, discharge, and transfer between healthcare institutions, or at least at critical points?				
Is medication reconciliation completed within a maximum of 24 hours after transition of care?				
Are procedures in place to manage medication processes in the event of information system failures?				
**POLICIES AND PROCEDURES**				
	1	2	3	4
Does the institution have a written policy, plan, or procedure for medication system safety?				
Is there a written procedure for safe medication prescribing (e.g., treatment duration, dose, route of administration)?				
Are there written procedures for the safe storage of medications (e.g., identification, safes, refrigerators)?				
Are there written procedures for the safe dispensing and distribution of medications?				
Is there a written procedure for the safe preparation and administration of medications?				
Are internal audits implemented for processes related to safe medication practices?				

MEDICATION PRESCRIBING SAFETY SCALE

Response scale:
1 – Does not exist / Implementation not planned
2 – Does not exist / Implementation planned within 3–6 months
3 – Exists / Implemented in some services
4 – Exists / Implemented throughout the institution

**POLICIES, PROCEDURES AND SAFE PRACTICES**				
	1	2	3	4
Is there a written standard requiring identification of drug name, dose, route, and administration schedule when home medication is prescribed?				
Is off-label medication prescribing required to be explicitly identified?				
Is there an induction programme with specific training for new physicians before independent prescribing?				
Are physicians required to perform medication reconciliation at critical transitions of care?				
Are awareness campaigns conducted to encourage physicians to inform patients about prescribed or modified medications?				
**ELECTRONIC PRESCRIBING**				
	1	2	3	4
Does oral medication prescribing always include instructions regarding preferred administration (e.g., fasting, after meals)?				
Does the electronic system automatically display dilution and infusion time for intravenous medications?				
Are there automatic alerts for drug allergies?				
Are there automatic alerts for patients prescribed anticoagulants or with hypocoagulation?				
Are there automatic alerts for maximum dose limits?				
Are there alerts related to laboratory values for dose-adjusted or potentially toxic medications?				
Are clinical decision support algorithms available for medication prescribing?				
**COMMUNICATION AND INFORMATION MANAGEMENT**				
	1	2	3	4
Is an electronic prescribing system in place?				
Is there a written standard requiring legible handwriting when electronic prescribing is not available?				
Is there a strategy to manage illegible prescriptions for LASA medications when electronic prescribing is unavailable?				
Are prescribing protocols implemented for emergency situations or when no physician is present?				
Is there a written standard prohibiting verbal or telephone medication orders, except in emergencies?				

Is there a standard requiring mandatory reporting of adverse drug reactions to the national pharmacovigilance system?				

MEDICATION SAFETY IN HOSPITAL PHARMACY SCALE

Response scale:
1 – Does not exist / Implementation not planned
2 – Does not exist / Implementation planned within 3–6 months
3 – Exists / Implemented in some services
4 – Exists / Implemented throughout the institution

**LABELLING AND STORAGE**				
	1	2	3	4
Are warning labels used to distinguish different dosages of the same medication?				
Are mechanisms in place to control medication expiry dates?				
Do pharmacy labels include minimum specifications such as active substance, dosage, expiry date, and batch number?				
Are narcotics stored in a dedicated, locked location with restricted access?				
Are high-risk medications identified with alert signage and stored in specific locations?				
Do pharmacy professionals verify patient identification, medication name, dose, route, frequency, and administration time before dispensing?				
Is there a written standard for medication storage and organization in clinical areas?				
Are medications stored according to expiry date, ensuring first-expiry–first-out use?				
Are refrigerated medications stored at 2–8°C with temperature monitoring and recording systems?				
**TRAINING AND SAFE PRACTICES**				
	1	2	3	4
Is medication checked by sampling each day by a second pharmacy professional before leaving the hospital pharmacy?				
Are dispensing errors recorded in the hospital pharmacy when they occur?				
Are identified dispensing errors reported in the incident reporting system, even if detected before leaving the pharmacy?				
Are awareness campaigns conducted to encourage returning unused medications to the pharmacy rather than placing them in stock?				

Does the hospital pharmacy disseminate information about new and existing medications to all professionals involved in medication processes?				
Is there an induction programme with specific training for new pharmacy staff before independent practice?				
**POLICIES AND PROCEDURES**				
	1	2	3	4
Do pharmacy services conduct awareness campaigns encouraging professionals to report LASA medications not included in the institutional list?				
Are trigger tools used to identify potential medication-related adverse events?				
Is there a procedure for separate storage and distinctive labelling of LASA medications?				
Do pharmacists/pharmacy technicians collaborate in medication reconciliation at critical transitions of care?				
Are there procedures for medication dispensing and distribution in the hospital pharmacy?				
Is the Pharmacy and Therapeutics Committee and/or medication safety officer involved in medication selection?				
Is there a policy for acquiring unit-dose or prefilled medications to minimize manipulation by pharmacy services?				
**DISPENSING AND DISTRIBUTION**				
	1	2	3	4
Is there a unit-dose or patient-specific medication distribution system?				
Are medications always dispensed in unit-dose packaging?				

MEDICATION PREPARATION AND ADMINISTRATION SAFETY SCALE

Response scale:
1 – Does not exist / Implementation not planned
2 – Does not exist / Implementation planned within 3–6 months
3 – Exists / Implemented in some services
4 – Exists / Implemented throughout the institution

**POLICIES AND PROCEDURES**				
	1	2	3	4
Are adequate resources available for nurses to prepare and administer medications without time pressure?				
Is there a policy prohibiting interruptions during medication preparation and administration?				
Is there a policy discouraging transcription of medication prescriptions?				
Is there a policy for acquiring standardized infusion pumps and syringe drivers?				
Is there a written standard requiring independent double-checks when dose calculations or dilutions are required?				
Are awareness campaigns conducted to encourage nurses to inform patients about medications being administered?				
Is there a written standard requiring nurses not to follow incomplete or unclear prescriptions?				
Is there a written standard prohibiting medication administration when doubts exist?				
Is there a written standard requiring double-checks for high-risk medications?				
**SAFE PRACTICES**				
	1	2	3	4
Is there an induction programme with specific training for new nurses before independent practice?				
Is there a written standard requiring verification of patient identification using at least two identifiers?				
Is positive patient identification performed whenever possible before medication administration?				
Is there a written standard requiring careful reading of medication labels before preparation and administration?				
Is medication prepared immediately before administration and one medication at a time?				
Is medication administration documented immediately and reasons recorded when not administered?				
**WORK ENVIRONMENT**				
	1	2	3	4

Are physical conditions adequate for safe medication preparation and administration?				
Is there a dedicated, restricted area for medication verification and preparation?				
Is there a policy discouraging printing of electronic prescriptions for medication preparation?				
Are drug dilution and infusion rate charts available and visible in preparation areas?				
Is there a written standard requiring nurses to educate patients and families about medication regimens and error prevention?				
Is there a system preventing nursing students from preparing or administering medications without supervision?				
Are awareness campaigns conducted to encourage nurses to seek clarification from other professionals when in doubt?				
Do nurses collaborate in medication reconciliation at critical transitions of care?				
**AVAILABLE RESOURCES**				
	1	2	3	4
Are automatic allergy alerts available in the medication administration system?				
Are enteral-only syringes implemented that cannot connect to intravenous systems?				
Are enteral feeding systems implemented that prevent connection to intravenous routes?				
Is there a Medication Administration Safety Checklist including the five rights of medication administration?				

MEDICATION SAFETY IN PATIENT INFORMATION AND EDUCATION SCALE

Response scale:
1 – Does not exist / Implementation not planned
2 – Does not exist / Implementation planned within 3–6 months
3 – Exists / Implemented in some services
4 – Exists / Implemented throughout the institution

	1	2	3	4
Are there campaigns to promote patient involvement in their own safety with regard to the safe use of medications?				
Are there initiatives to disseminate information to patients and families about the safe use of medications?				
Are awareness campaigns conducted to encourage patients and families to ask questions about prescribed and administered medications, medication regimen management, and error prevention?				
Are awareness campaigns conducted to encourage patients to inform physicians and nurses about any allergies and medications they are currently taking?				
Is there a system and/or tools in place to facilitate communication between different healthcare providers, ensuring that patients' outpatient medications are accurately transmitted at admission and discharge?				

Thank you for your collaboration
